# Factors associated with abdominal obesity in children

**DOI:** 10.1016/j.rpped.2015.04.002

**Published:** 2015

**Authors:** Matheus Ribeiro Theodósio Fernandes Melzer, Isabella Mastrangi Magrini, Semíramis Martins Álvares Domene, Paula Andrea Martins

**Affiliations:** a Universidade Federal de São Paulo (Unifesp), Campus Baixada Santista, Santos, SP, Brazil

**Keywords:** Nutritional epidemiology, Children, Abdominal obesity, Waist circumference

## Abstract

**Objective:**

To identify the association of dietary, socioeconomic factors, sedentary behaviors and maternal nutritional status with abdominal obesity in children.

**Methods:**

A cross-sectional study with household-based survey, in 36 randomly selected census tracts in the city of Santos, SP. 357 families were interviewed and questionnaires and anthropometric measurements were applied in mothers and their 3-10 years-old children. Assessment of abdominal obesity was made by maternal and child's waist circumference measurement; for classification used cut-off points proposed by World Health Organization (1998) and Taylor et al. (2000) were applied. The association between variables was performed by multiple logistic regression analysis.

**Results:**

30.5% of children had abdominal obesity. Associations with children's and maternal nutritional status and high socioeconomic status were shown in the univariate analysis. In the regression model, children's body mass index for age (OR=93.7; 95%CI 39.3-223.3), female gender (OR=4.1; 95%CI 1.8-9.3) and maternal abdominal obesity (OR=2.7; 95%CI 1.2-6.0) were significantly associated with children's abdominal obesity, regardless of the socioeconomic status.

**Conclusions:**

Abdominal obesity in children seems to be associated with maternal nutritional status, other indicators of their own nutritional status and female gender. Intervention programs for control of childhood obesity and prevention of metabolic syndrome should consider the interaction of the nutritional status of mothers and their children.

## Introduction

The worldwide obesity epidemic is increasing at an alarming rate in childhood and can be observed in developing countries, which have shown an increase in the prevalence of childhood obesity in recent decades.^1^ In Brazil, a study with a sample of children aged 7-10 years showed a prevalence of overweight and obesity of 26.7% for boys and 34.6% for girls.^2^

As a consequence of excess weight, abdominal obesity is associated with cardiovascular risk factors and metabolic disorders, which may already be present in childhood.^3^^,^ 4 Abdominal obesity is understood as the accumulation of fat in the abdominal region assessed by an anthropometric and/or body composition measure that shows a value above a specific and sensitive cutoff point.^4^ Among the methods used for the diagnosis, waist circumference (WC), widely used in the assessment of nutritional status in adults, has been also used in children.^4^^,^ 5 Studies with different populations have proposed distributions in percentiles and cutoffs for WC in children, but there is still no consensus about the criteria used for the assessment of this group.^4^

The accuracy of the WC measurement when compared to other methods of nutritional status assessment in children, such as the body mass index (BMI) and the waist/height ratio (WHtR), was evaluated in studies of which results showed the use of this measure in high blood pressure risk identification in combination with BMI or as a factor associated with dyslipidemia and hyperglycemia.^6^^,^ 7

Some factors associated with excess weight and abdominal obesity in children, described in the scientific literature, are: the family's socioeconomic status,^8^ parents’ nutritional status,^9^ and children's sedentary behaviors.^10^ It is also known that unhealthy eating habits and high intake of macronutrients are possible causes of abdominal obesity.^3^ However, few studies have employed the WC to determine abdominal fat in Brazilian children as the outcome of interest, and to investigate the possible associated factors. The aim of this study is to assess the association of dietary and socioeconomic factors, sedentary behaviors and maternal nutritional status with abdominal obesity in children aged 3-10 years in Santos city, state of São Paulo, Brazil.

## Method

This study is part of the research project "Nutritional Environment Assessment in the city of Santos" (AMBNUT), approved by the Institutional Review Board of Universidade Federal de São Paulo (Processes: 275/2009 and 276/2009) and funded by Fundação de Amparo a Pesquisa do Estado de São Paulo (FAPESP) (process n.: 2009/01361-0). This was a cross-sectional, household-based project, carried out from January to December 2010, when two visits were made to the households to collect socioeconomic and anthropometric data, as well as information on the families’ health and food habits.

According to data from the Brazilian Institute of Geography and Statistics (Instituto Brasileiro de Geografia e Estatística - IBGE), also published in 2010, the municipality had 419,400 residents living in an area of 281,000 km^2^. The insular portion of the municipality is divided into four administrative regions: Coast, Central, Northwest and Hills; the Coastal region is characterized by higher income compared to the others. It is estimated that 55% of families live in the Coastal region, 11% in the Central region, 25% in the Northwest and 9% in the Hills.

The AMBNUT project sample consisted of 538 families from 36 of the 566 census sectors in the insular part of the municipality, randomly selected proportionally to the population residing in three of the four regions: Central, Northwest and Coastal regions. The Hills region was not included due to difficulties in accessing the place. Thus, 29 sectors were assessed in the Coastal region, three sectors in the Central region and four sectors in the Northwest region, redistributing the proportion of residents in the Hills’ regions to the other regions.

Sample calculation considered a prevalence of excess weight in children younger than five years of 7% in the Southeast region, measured by the National Demographic and Health Survey (PNDS), using a significance level of 5% and 80% test power for a two-tailed test, with a loss of 10%.

For data collection, six interviewers, both graduated and undergraduate students from the health area, were trained to apply the questionnaire and perform the fieldwork, and worked in pairs. The training was carried out by the research team responsible for the project, with a 40-h duration, which included training in laboratory and supervised monitoring during the initial field activities.

The enrollment of each sector was carried out to identify eligible households, having as inclusion criteria homes that had at least one child younger than ten years living with the birth mother. If the mother had any disorder that could affect her nutritional status (cancer, AIDS or infectious diseases) or had undergone bariatric surgery the pair was excluded. If the household had more than one child in the age group being assessed, the participant was chosen by drawing lots. The data collection procedures were carried out only after the children's mothers signed the Informed Consent Form. The response rate of assessed households at enrollment was 70%, and the mean duration of interviews was 100 min.

A total of 357 pairs of mothers and children were considered eligible for this specific study, corresponding to households with children aged three years old or older, in which the WC measure was collected. This criterion maintained the assessed sample representativeness, and the power of the association test was 90%.

Children's food intake was estimated using two 24-h recalls, in interviews with the mother, one applied at each visit. The nutritional composition of the food was calculated using the Avanutri^®^ program v.4.0 (Avanutri & Nutrição Serviços e Informática Ltda., Três Rios, Brasil); the system database was expanded using data from the Brazilian Table of Food Composition (TACO) and the United States Department of Agriculture (USDA).

The median consumption of each macronutrient was used as a cutoff point for the analysis, taking into account the children's age (3 years, 4-8 years and 9-10 years) to generate the variable. This categorization includes the age groups for which the Institute of Medicine has specific recommendations for each nutrient according to the Dietary Reference Intake (DRI), in an attempt to not underestimate or overestimate consumption.

The weight and height of mothers and children were collected using a Tanita^®^ portable digital scale and Alturexata^®^ portable stadiometer, following the standardized techniques established by Lohman et al.^11^Additionally, the mothers’ tricipital, bicipital, suprailiac and subscapular skinfolds were collected in triplicate, to calculate the mean value and estimate body fat percentage.

WC was measured in duplicate using a 150-cm inelastic metric tape, with measurement being standardized at the midpoint between the last rib and the iliac crest, with the child or the mother in the standing position, without clothes covering the abdominal region; the reading was performed at expiration.

The proposal by Taylor et al. was used to evaluate children's abdominal fat, which considers values above the 80th percentile (p80) as abdominal obesity.^12^ As for BMI, the World Health Organization (WHO) curves were used to identify excess weight.^13^

To classify maternal nutritional status, BMI and WC were used according to the WHO recommendations, which depict BMI≥25kg/m^2^ as excess weight and WC≥80cm as abdominal obesity.^14^ Body fat classification was considered when values were ≥32%, indicating elevated body fat.^11^

The children's sedentary behaviors were assessed based on the time spent watching TV, using the computer and walking, as well as bicycle riding as a main transport mode for daily activities or as leisure activities, according to the YOUTH validated questionnaire of the Study Center of the Physical Fitness Laboratory of São Caetano do Sul (CELAFICS).^15^ The analysis used a cutoff of 2h of watching TV daily, as recommended by the American Academy of Pediatrics^16^; the same criterion was applied to computer screen time use.

The socioeconomic assessment of families was carried out using the IBGE and PNDS questionnaires, which assess characteristics of households, schooling and income. For the stratification of families, the Brazilian Economic Classification Criteria were used, as proposed by the Brazilian Association of Research Companies (ABEP).^17^ For the analyses, classes were grouped into A+B (high socioeconomic status) and C+D+E (low socioeconomic status).

Descriptive statistical analysis of the sample was performed, stratified by the children's age group. Associations between the variables of interest and abdominal obesity were verified using multiple logistic regression models, with WC>p80 being the outcome, and socioeconomic variables, maternal nutritional status and the child's individual variables being used to adjust the model. Initially, the chi-square test was used for the univariate analysis and variables with *p*<0.20 were included in the multivariate analysis. In the final model, only the variables with *p*-value <0.05 remained. The Hosmer-Lemeshow test was used to verify the goodness-of-fit. These results are shown with the values of odds ratio (OR) and 95% confidence interval (95%CI).

Epi Info^®^ software v.3.5 (CDC, Atlanta, GA, USA) was used for dietary data computation. The *Z*-score values were calculated using the Anthro Plus^®^ program, v.1.0.2 (WHO, Geneva, Switzerland). Analyses were performed using the Statistical Package for the Social Sciences^®^ (SPSS) software v.16 (SPSS Inc., Chicago, IL, USA).

## Results

Tables 1 and 2 disclose the descriptive sample data according to the prevalence of assessed factors. Overall, it was observed that 30.5% of children and 64% of mothers had abdominal obesity. The univariate analysis shown in Table 3 indicated an association between having abdominal obesity and excess weight according to BMI/age. There were no significant associations with the consumption of nutrients for carbohydrates, lipids and protein; however, protein intake was subsequently tested in the regression model (*p*=0.085). None of the variables related to sedentary behavior showed an association with the outcome (*p*>0.20). Significant associations were observed between the variables of social stratification according to ABEP, car ownership, maternal work outside the home, maternal excess weight, and maternal total and central obesity (Table 3).

**Table 1:** Prevalence of the sample's individual variables, according to the children's age group. Santos, 2012 (n = 357).

	3–4 years			5–7 years			8–10 years			Total	
	*n*	%		*n*	%		*n*	%		*n*	%
**Children's characteristics**											
*Gender*											
Male	50	58.8		68	52.7		75	52.4		193	54.1
Female	35	41.2		61	47.3		68	47.6		164	45.9

*Abdominal obesity*											
Yes	32	37.6		28	21.7		49	34.3		109	30.5
No	53	62.4		101	78.3		94	65.7		248	69.5

*Excess weight according to BMI/age*											
Yes	12	14.1		39	30.2		62	43.4		113	31.7
No	73	85.9		90	69.8		81	56.6		244	68.3

*Time spent watching TV*											
High	73	85.9		108	83.7		116	81.1		297	83.2
Adequate	12	14.1		21	16.3		27	18.9		60	16.8

*Time spent using computer*											
High	6	7.1		28	21.7		41	28.7		75	21.0
Adequate	79	92.9		101	78.3		102	71.3		282	79.0

*Walking as the main transport mode*											
Yes	48	56.5		84	65.1		91	63.6		223	62.5
No	37	43.5		45	34.9		52	36.4		134	37.5

*Bicycle as the main transport mode*											
Yes	4	4.7		11	8.5		12	8.4		27	7.6
No	81	95.3		118	91.5		131	91.6		330	92.4

*Carbohydrate consumption* a											
Higher than/same as median	38	46.3		42	33.4		41	28.8		121	34.5
Below median	44	53.7		84	66.6		101	71.2		229	65.5

*Protein consumption* a											
Higher than/same as median	44	53.6		57	45.2		72	50.7		173	49.4
Below median	38	46.4		69	54.8		70	49.3		177	50.6

*Lipid consumption* a											
Higher than/same as median	49	59.7		54	42.8		67	47.1		170	48.5
Below median	33	40.3		72	57.2		75	52.9		180	51.5

BMI, body mass index.

a *n* = 350.

**Table 2:** Prevalence of maternal and socioeconomic variables of the sample, according to the children's age group. Santos, 2012 (*n* = 357).

	3–4 years			5–7 years			8–10 years			Total	
	*n*	%		*n*	%		*n*	%		*n*	%
**Maternal characteristics**											
*Excess weight according to BMI* a											
Yes	35	41.2		69	53.5		88	61.9		192	53.9
No	50	58.8		60	46.5		54	38.1		164	46.1

*Body fat* b											
High	26	30.6		34	26.9		26	18.8		86	24.6
Adequate	59	69.4		92	73.1		112	81.2		263	75.4

*Abdominal obesity* a											
Yes	48	56.5		79	61.2		101	71.1		228	64
No	37	43.5		50	38.8		41	28.9		128	36

**Socioeconomic characteristics**											
*Socioeconomic level (ABEP)*											
High	51	60		75	58.1		86	60.1		212	59.4
Low	34	40		54	41.9		57	39.9		145	40.6

*Car ownership*											
Yes	50	58.8		67	51.9		80	55.9		197	55.2
No	35	41.2		62	48.1		63	44.1		160	44.8

*Schooling*											
>High school	34	40		47	36.4		35	24.5		116	32.5
≤High school	51	60		82	63.6		108	75.5		241	67.5

*Work outside home*											
Yes	54	63.5		76	58.9		90	62.9		220	61.6
No	31	36.5		53	41.1		53	37.1		137	38.4

BMI, body mass index; ABEP, Brazilian Association of Research Companies (Associação Brasileira de Empresas de Pesquisa).

a *n* = 356.

b *n* = 349.

**Table 3:** Univariate analysis of exploratory variables and association with the children's abdominal obesity as the dependent variable. Santos, 2012 (*n* = 357).

	_crude_OR	95%CI	*p*-value
**Children's characteristics**			
*Gender*			
Male	1.0	–	0.172
Female	1.4	0.8–2.2	

*Excess weight according to BMI/age*			
Yes	62.4	30.1–129.2	<0.001
No	1.0	–	

*Protein consumption*			
Higher than/same as median	1.0	–	0.085
Below median	0.6	0.4–1.0	

**Maternal characteristics and socioeconomic level**			
*Socioeconomic level (ABEP)*			
High	1.0	–	0.017
Low	0.6	0.3–0.9	

*Car ownership*			
Yes	1.8	1.1–2.8	0.012
No	1.0	–	

*Schooling*			
>High school	1.0	–	0.171
≤High school	0.7	0.4–1.1	

*Work outside home*			
Yes	1.9	1.1–3.0	0.010
No	1.0	–	

*Excess weight (BMI)* a			
Yes	1.7	1.0–2.7	0.024
No	1.0	–	

*Body fat* b			
High	1.9	1.0–3.3	0.033
Adequate	1.0	–	

*Abdominal obesity* a			
Yes	2.2	1.3–3.6	0.002
No	1.0	–	

BMI, body mass index; ABEP, Brazilian Association of Research Companies (Associação Brasileira de Empresas de Pesquisa); OR, odds ratio; CI, confidence interval.

a *n* = 356.

b *n* = 349.

Table 4 shows the results of the final logistic regression model. In the final model, the association between the child's abdominal obesity and childhood excess weight according to BMI/age, female gender and the mothers’ abdominal obesity were significant. The ABEP socioeconomic classification variable remained as a control variable.

**Table 4:** Multiple logistic regression model with the children's abdominal obesity as the dependent variable. Santos, 2012 (*n* = 356).

	_Adjusted_OR	95%CI	*p*-value
*Excess weight according to the child's BMI/age*			
Yes	93.7	39.3–223.3	<0.001
No	1.0	–	

*Child's gender*			
Male	1.0	–	0.012
Female	4.1	1.8–9.3	

*Maternal abdominal obesity*			
Yes	2.7	1.2–6.0	0.01
No	1.0	–	

*Socioeconomic level according to ABEP*			
High	1.0	–	0.08
Low	0.5	0.2–1.0	

BMI, body mass index; ABEP, Brazilian Association of Research Companies (Associação Brasileira de Empresas de Pesquisa); OR, odds ratio; CI, confidence interval.

## Discussion

This study identified associations between the abdominal obesity in children and the child's nutritional status (excess weight according to BMI/age), the child's gender (female) and the mother's nutritional status (abdominal obesity).

The results showed a prevalence of 30.5% of children with abdominal obesity. A study with Brazilian children aged 7-10 years found a prevalence of 22% for girls and 26.5% for boys, slightly lower values, but estimated based on another reference.^18^ Another study identified a prevalence of 33.7% of excess weight in children from Santos city,^19^ values that are consistent with the data in this research. This scenario shows that excess weight and child abdominal obesity are both prevalent.

At the univariate analysis, child's risk for overweight or overweight was associated with the presence of abdominal obesity. This finding is consistent with the fact that the trunk region is one with increased susceptibility to fat accumulation. Although this variable remained in the final regression model, it is relevant to consider its effect's overestimation, given the marked association between excess weight and the assessed outcome; it is also important to consider the broad CI (OR=93.7, 95%CI: 39.3-223.3).^12^^,^ 20 A study with Brazilian adolescents produced similar results, when considering that individuals with a higher amount of subcutaneous fat were 133.6 times more likely to have abdominal obesity^21^; the authors used the classification by Taylor et al.,^12^ also used in the present study.

The results also show that girls are 4.1 times more likely to have abdominal obesity; one expects to find greater adiposity in females, taking into consideration the BMI^22^; however, an association has been shown between adiposity, BMI and WC in male children and adolescents.^21^^,^ 23 It is also known that the adult male has more visceral fat accumulation than adult women^24^; thus, it is worth considering the hypothesis that most abdominal fat found in girls can be a characteristic of this sample; i.e., it is possible that the typical conditions of body fat accumulation are more evident only after puberty, when considering that the sample's age range may be too young.

Activities considered as sedentary practices showed no association with abdominal obesity, which contradicts previous findings on the effect of time spent watching TV and active transport to school.^10^^,^ 22 ^,^ 23 A study with Brazilian adolescents also found a different result: an insufficient level of physical activity was associated with an elevated WC.^25^ It is possible that the study variables were not good markers of sedentary behaviors, which may bring health risks to this population. However, it should be considered that the time spent watching TV and using the computer was reported by the child's mother and may have been underestimated.

High protein intake was associated with abdominal obesity in the univariate analysis, but it subsequently lost significance in the regression model. The increase in protein consumption showed an opposite association: through changes in the diet, more protein generated a reduction of 2.7 cm in WC of children and adolescents in relation to a group with low protein intake, in a longitudinal study.^26^

Maternal nutritional status was significantly associated with abdominal obesity in children in all assessed parameters, especially with the WC measurement in the multivariate analysis. In our sample, a mother with abdominal obesity increases by 2.7-fold the chance of her children also developing this condition. Studies carried out in Mexico report that children of parents with abdominal obesity are 2.85 times more likely to have the same condition,^9^ a value similar to that found in our study. Other studies also found an association between excess weight in children and maternal obesity.^22^^,^ 23 This indicates that the nutritional status of both the child's mother and father may be related to this outcome, and it should be considered that such influence could be associated with both genetic and sociocultural factors of family habits. The nutritional attention care in maternal and child health thus should begin during the prenatal period and encompass the whole family structure. It has been shown that parental involvement in nutritional education interventions and in promoting physical activity for children beneficially assist in reducing BMI and other nutritional status parameters,^27^ and it should be previously considered that the parents’ perception of their child's nutritional status can be an obstacle, given the difficulty in identifying overweight and, therefore, recognizing the importance of including their child in such activities.^28^

Even though it had no significance in the final model, the univariate analysis showed a significant association between abdominal obesity in children and socioeconomic status by social stratification, car ownership and the fact that the mother worked out of the household. High socioeconomic level, represented by the type of school and town/city, has also been associated with abdominal obesity in Indian children.^8^ However, another study with Brazilian children younger than five years did not observe an association between socioeconomic status, also represented by the ABEP, and excess weight^29^; it can be suggested that other factors have a greater influence on nutritional status than socioeconomic level.

Car ownership could also be associated with physical activity, as parents who have a car would use it to take their children to daily activities, thus preventing them from riding the bicycle or walking, for convenience or for fear of urban violence.^10^ As for children whose mothers work outside the home, considering the fact that these mothers are not present during the day to prepare a balanced meal for their children, they eventually choose convenient and quick options to feed them, habits that can be later incorporated by the children.

The study of food intake has limitations, such as the small number of 24-h recalls and obtaining data through interviews with the mother, which can result in underreporting. Additionally, considering this study applied an extensive investigation questionnaire, some questions and measures could not be applied or measured by the interviewers due to alleged discomfort or refusal on the part of the respondent and, thus, there are some variables with missing values. The response rate of 70% may also have affected the results of some associations. Moreover, considering this is a cross-sectional study, one cannot establish a causality association between the analyzed factors. In spite of these limitations, the contributions of this study include the observation that maternal nutritional status, the occurrence of excess weight and female gender are associated with abdominal obesity in children, regardless of their socioeconomic status.

Metabolic syndrome in children is still an emerging topic, for which different clinical aspects are considered in the diagnosis, depending on the theoretical reference.^30^ Elevated WC is one of these aspects and, when compared to others, such as serum levels of glucose and lipids, it has advantages for being a noninvasive, easy to measure procedure. Thus, its inclusion in routine primary care would help to plan interventions and interdisciplinary activities associated with the fight against chronic diseases in childhood.

The associations involving the child's WC are still uncertain. More studies in this line of research are needed to validate some of the assumptions made herein. It is expected that this study will contribute to the development of public policies for the prevention of chronic diseases, considering the importance of nutritional education actions directed at the family environment, aiming to result in a greater impact on the nutritional status of children.
