# Supplementary material for: Factors associated with abdominal obesity in children
Source: Rev Paul Pediatr. 2015 Oct-Dec;33(4):437–44. doi: 10.1016/j.rpped.2015.04.002 (PMC4685564; doi:10.1016/j.rpped.2015.04.002)
Supplement: Translation

# Translation: Fatores associados ao acúmulo de gordura abdominal em crianças

**Authors:** Matheus Ribeiro Theodósio Fernandes Melzer, Isabella Mastrangi Magrini, Semíramis Martins Álvares Domene, Paula Andrea Martins

**Affiliations:** a Universidade Federal de São Paulo (Unifesp), Campus Baixada Santista, Santos, SP, Brasil

**Keywords:** Epidemiologia nutricional, Crianças, Gordura abdominal

## Abstract

**Objetivo:**

Identificar fatores individuais (dietéticos, comportamento sedentário) e familiares (estado nutricional materno e nível socioeconômico) associados com o acúmulo de gordura abdominal de crianças.

**Métodos:**

Estudo de delineamento transversal de base domiciliar, em 36 setores censitários sorteados aleatoriamente na cidade de Santos/SP. Foram entrevistadas 357 famílias para aplicação de questionários e aferição de medidas antropométricas em mães e crianças de 3-10 anos. A avaliação do acúmulo de gordura abdominal foi feita pela medida da circunferência da cintura de mães e crianças com o uso da recomendação da Organização Mundial da Saúde (1998) e a proposta de Taylor et al. (2000), respectivamente. A associação entre as variáveis foi verificada por meio de regressão logística múltipla.

**Resultados:**

Verificou-se que 30,5% das crianças apresentaram acúmulo de gordura abdominal. Na análise univariada, o acúmulo de gordura abdominal esteve associado ao estado nutricional materno e da criança e ao nível socioeconômico elevado. Na análise multivariada, foram observadas associações com excesso de peso pelo índice de massa corporal para idade (OR=93,7; IC95% 39,3-23,3); ser do sexo feminino (OR=4,1; IC95% 1,8-9,3) e acúmulo de gordura abdominal materno (OR=2,7; IC95% 1,2-6); independentemente do nível socioeconômico.

**Conclusões:**

O acúmulo de gordura abdominal em crianças mostrou-se associado ao estado nutricional materno, aos indicadores de seu próprio estado nutricional e ao sexo feminino. Programas de intervenção para controle da obesidade infantil e prevenção da síndrome metabólica relacionada ao acúmulo de gordura abdominal devem levar em consideração a interação do estado nutricional de mães e seus filhos.

## Introdução

A epidemia mundial da obesidade vem crescendo em proporções alarmantes na infância, o que pode ser observado em países em desenvolvimento, que apresentaram aumento na prevalência da obesidade infantil, nas últimas décadas.^1^ No Brasil, estudo com amostra de crianças de 7 a 10 anos apontou uma prevalência de sobrepeso e obesidade de 26,7% para meninos e 34,6% para meninas.^2^

Consequente ao excesso de peso, o acúmulo de gordura na região abdominal está associado a fatores de risco cardiovascular e a distúrbios metabólicos, que podem estar presentes já na infância.^3^^,^ 4 Compreende-se como acúmulo de gordura na região abdominal um excesso de gordura avaliado por uma medida antropométrica e/ou de composição corporal que apresente um valor acima de um ponto de corte específico e sensível.^4^ Entre os métodos para esse diagnóstico, a circunferência da cintura (CC), bastante usada na avaliação do estado nutricional de adultos, também vem sendo usada em crianças.^4^^,^ 5 Estudos com diferentes populações já propuseram distribuições em percentis e pontos de corte para a CC de crianças, porém ainda não há consenso sobre os critérios para avaliação desse grupo.^4^

A acurácia da CC em comparação com outros métodos de diagnóstico do estado nutricional da criança, como o Índice de Massa Corporal (IMC) e a razão cintura/altura, foi avaliada em estudos cujos resultados apontam aplicações dessa medida para auxiliar na identificação de risco de pressão arterial elevada em combinação ao IMC ou como fator associado à dislipidemia e à hiperglicemia.^6^^,^ 7

Alguns fatores associados ao excesso de peso e acúmulo de gordura abdominal em crianças, descritos na literatura científica, são: nível socioeconômico da família,^8^ estado nutricional dos pais^9^ e comportamentos sedentários da criança.^10^ Sabe-se também que hábitos alimentares não saudáveis e o consumo elevado de macronutrientes são apontados como possíveis causas do acúmulo de gordura abdominal.^3^ Entretanto, poucos estudos empregaram a CC para determinar a gordura abdominal em crianças brasileiras como desfecho de interesse e para investigar quais os possíveis fatores associados. O objetivo deste estudo é analisar a associação de fatores dietéticos, socioeconômicos, comportamentos sedentários e do estado nutricional materno com o acúmulo de gordura abdominal de crianças de 3-10 anos no município de Santos.

## Método

Esse estudo está inserido no projeto de pesquisa Avaliação do Ambiente Nutricional no Município de Santos (Ambnut) aprovado no Comitê de Ética em Pesquisa da Universidade Federal de São Paulo (Processos: 275/2009 e 276/2009) e financiado pela Fundação de Amparo a Pesquisa do Estado de São Paulo (processo n° 2009/01361-0). O projeto teve delineamento transversal de base domiciliar; de janeiro a dezembro de 2010 foram feitas duas visitas para investigar dados socioeconômicos, antropométricos, sobre saúde e hábito alimentar das famílias.

De acordo com dados do Instituto Brasileiro de Geografia e Estatística (IBGE), também publicados em 2010, o município apresentava 419.400 residentes em uma área de 281.000 km^2^. A parte insular da cidade é dividida em quatro regiões administrativas: Orla, Centro, Noroeste e Morros; a região da Orla é caracterizada por maior poder aquisitivo em comparação com as demais. Estima-se que 55% das famílias residam na região da Orla, 11% no Centro, 25% na Zona Noroeste e 9% nos Morros.

A amostra do projeto Ambnut foi de 538 famílias, provenientes de 36 dos 566 setores censitários da parte insular do município, sorteados aleatoriamente de forma proporcional à população moradora de três das quatro regiões: Centro, Noroeste e Orla. A região dos Morros não foi incluída por dificuldades de acesso. Dessa forma, 29 setores foram investigados na Orla, três setores no Centro e quatro setores na região Noroeste e redistribuiu-se a proporção de residentes dos Morros às demais regiões.

O cálculo amostral considerou a prevalência de sobrepeso em crianças menores de cinco anos de 7% na região Sudeste, avaliada pela Pesquisa Nacional de Demografia e Saúde (PNDS), adotando nível de significância de 5% e poder de teste de 80% para um teste bicaudal, com perda de 10%.

Para coleta de dados, seis entrevistadores graduados e graduandos da área de saúde foram treinados para a aplicação do questionário e trabalho de campo, organizados em duplas. O treinamento foi conduzido pela equipe de pesquisadores responsáveis pelo projeto, com carga horária de 40 horas, que compreendia treinamento em laboratório e acompanhamento supervisionado às primeiras atividades em campo.

Foi feito o arrolamento de cada setor, com o objetivo de identificar domicílios elegíveis, que teve como critérios de inclusão aquele em que residia pelo menos uma criança menor de dez anos juntamente com sua mãe biológica, desde que a mesma não relatasse qualquer distúrbio que pudesse afetar seu estado nutricional (câncer, Aids ou doenças infecciosas) ou tivesse feito cirurgia bariátrica. Caso houvesse no domicilio mais de uma criança na faixa etária a ser avaliada, fazia-se sorteio. Os procedimentos da coleta foram feitos somente após as mães das crianças assinarem o Termo de Consentimento Esclarecido. A taxa de resposta dos domicílios investigados no arrolamento foi de 70% e o tempo médio de entrevistas foi de 100 minutos.

Para este estudo específico, foram considerados elegíveis 357 pares de mães e filhos, o que correspondeu aos domicílios com crianças de três ou mais anos, nos quais a medida da CC era coletada. Esse recorte manteve a representatividade da amostra investigada e o poder de teste de associação foi de 90%.

A ingestão alimentar das crianças foi estimado por meio de dois Recordatórios de 24 horas, em entrevistas com a mãe, um aplicado em cada visita. A composição nutricional dos alimentos foi calculada por meio do programa Avanutri^®^ v.4.0 (Avanutri & Nutrição Serviços e Informática Ltda., Três Rios, Brasil); o banco do sistema foi ampliado com dados da Tabela Brasileira de Composição de Alimentos (Taco) e do United States Department of Agriculture (USDA).

Foi usada para análise a mediana do consumo de cada macronutriente, como ponto de corte, e levou-se em conta a faixa etária das crianças (3 anos; 4-8 anos e 9-10 anos) para geração da variável. Essa categorização compreende as faixas etárias nas quais o Institute of Medicine tem uma recomendação específica para cada nutriente pela Dietary Reference Intake (DRI), na tentativa de não subestimar ou superestimar o consumo.

Foram coletados o peso e altura das mães e crianças com o uso de balança digital portátil Tanita^®^ e estadiômetro móvel Alturexata^®^, com as técnicas padronizadas por Lohman et al.^11^ Com as mães, foram coletadas dobras cutâneas triciptal, biciptal, suprailíaca e subescapular em triplicata para cálculo do valor médio e estimativa do percentual de gordura corporal.

A CC foi aferida, em duplicata, com uma fita métrica não extensível de 150 cm. Sua aferição foi padronizada no ponto médio entre a última costela e a crista ilíaca, com a criança ou a mãe de pé, sem roupa que cobrisse a região abdominal; a leitura foi feita no momento da expiração.

Para avaliação da gordura abdominal infantil, considerou-se a proposta por Taylor et al., que considera valores acima ao percentil 80 (p80) como acúmulo de gordura abdominal.^12^ Para o IMC, consideraram-se as curvas da Organização Mundial da Saúde (OMS), para identificação do excesso de peso.^13^

Para classificar o estado nutricional materno, adotaram-se o IMC e a CC de acordo com a recomendação da OMS, que traz valores de IMC≥25kg/m^2^como excesso de peso e CC≥80cm como acúmulo de gordura abdominal.^14^ Para classificar o acúmulo de gordura corporal consideraram-se os valores ≥32% como indicativos de gordura corporal elevada.^11^

Os comportamentos sedentários das crianças foram avaliados a partir do tempo gasto assistindo a TV, tempo com uso do computador e caminhada, além do uso de bicicleta como meio de transporte principal para as atividades diárias ou como lazer, conforme questionário validado Youth do Centro de Estudos do Laboratório de Aptidão Física de São Caetano do Sul (Celafics).^15^ Para as análises, foi considerado como ponto de corte o valor de duas horas assistindo a TV por dia, segundo recomendação da American Academy of Pediatrics;^16^ o mesmo critério foi aplicado para tempo de uso de computador.

Para avaliação socioeconômica das famílias foram usados questionários do IBGE e da PNDS, que avaliam características dos domicílios, escolaridade e renda. Para a estratificação das famílias, empregou-se o Critério de Classificação Econômica Brasil proposto pela Associação Brasileira de Empresas de Pesquisa (Abep).^17^ Para análises, as classes foram agrupadas em A + B (alto nível socioeconômico) e C + D + E (baixo nível socioeconômico).

Foi feita análise estatística descritiva da amostra, estratificada pela faixa etária das crianças. Associações entre as variáveis de interesse e o acúmulo de gordura abdominal foram verificadas com modelos de análise de regressão logística múltipla, sendo a CC acima do p80 como desfecho e variáveis socioeconômicas, estado nutricional materno e as variáveis individuais da criança para ajuste no modelo. Inicialmente foi usado o teste do qui-quadrado para a análise univariada e as variáveis com valor de *p*<0,20 foram incluídas na análise multivariada. No modelo final, permaneceram as variáveis com valor de *p*<0,05. Foi usado o teste de Hosmer-Lemeshow para verificar o ajuste do modelo. Esses resultados são apresentados com os valores de *odds ratio* (OR) e Intervalo de Confiança (IC) de 95%.

Para computação dos dados dietéticos foi usado o *software* Epi Info^®^ v.3.5 (CDC, Atlanta, GA, EUA). Os valores de escore-Z foram calculados com o programa Anthro Plus^®^ v.1.0.2. (WHO, Genebra, Suíça). As análises foram feitas no *software*Statistical Package for the Social Sciences^®^ (SPSS) v.16 (SPSS Inc., Chicago, IL, EUA).

## Resultados

Nas Tabelas 1 e 2 são apresentados os dados descritivos da amostra, pela prevalência dos fatores avaliados. No total, observou-se que 30,5% das crianças e 64% das mães apresentaram acúmulo de gordura abdominal. A análise univariada apresentada na Tabela 3 indicou associação entre apresentar acúmulo de gordura abdominal e excesso de peso pelo IMC/Idade. Não foram encontradas associações significativas com o consumo de nutrientes para carboidratos, lipídios e proteína; no entanto, o consumo de proteína foi testado no modelo de regressão posteriormente (*p*=0,085). Todas as variáveis relacionadas aos comportamentos sedentários igualmente não demonstraram associação com o desfecho (*p*>0,20). Foram encontradas associações significativas entre as variáveis de estratificação social pela Abep, posse de carro, trabalho materno fora de casa, excesso de peso materno, acúmulo de gordura total e central materna (Tabela 3).

**Tabela 1:** Prevalência das variáveis individuais das crianças da amostra, de acordo com a faixa etária das crianças. Santos, 2012 (n=357)

	3‐4 anos			5‐7 anos			8‐10 anos			Total	
	n	%		n	%		n	%		n	%
**Características das crianças**											
*Sexo*											
Masculino	50	58,8		68	52,7		75	52,4		193	54,1
Feminino	35	41,2		61	47,3		68	47,6		164	45,9
*Acúmulo de gordura abdominal*											
Sim	32	37,6		28	21,7		49	34,3		109	30,5
Não	53	62,4		101	78,3		94	65,7		248	69,5
*Excesso de peso pelo IMC/idade*											
Sim	12	14,1		39	30,2		62	43,4		113	31,7
Não	73	85,9		90	69,8		81	56,6		244	68,3
*Tempo assistindo a televisão*											
Elevado	73	85,9		108	83,7		116	81,1		297	83,2
Adequado	12	14,1		21	16,3		27	18,9		60	16,8
*Tempo em uso de computador*											
Elevado	6	7,1		28	21,7		41	28,7		75	21,0
Adequado	79	92,9		101	78,3		102	71,3		282	79,0
*Caminhada como transporte principal*											
Sim	48	56,5		84	65,1		91	63,6		223	62,5
Não	37	43,5		45	34,9		52	36,4		134	37,5
*Bicicleta como transporte principal*											
Sim	4	4,7		11	8,5		12	8,4		27	7,6
Não	81	95,3		118	91,5		131	91,6		330	92,4
*Consumo de carboidratos* a											
Acima/igual a mediana	38	46,3		42	33,4		41	28,8		121	34,5
Abaixo da mediana	44	53,7		84	66,6		101	71,2		229	65,5
*Consumo de proteína* a											
Acima/igual a mediana	44	53,6		57	45,2		72	50,7		173	49,4
Abaixo da mediana	38	46,4		69	54,8		70	49,3		177	50,6
*Consumo de lipídios* a											
Acima/igual a mediana	49	59,7		54	42,8		67	47,1		170	48,5
Abaixo da mediana	33	40,3		72	57,2		75	52,9		180	51,5

IMC, Índice de Massa Corporal.

a n=350.

**Tabela 2:** Prevalência das variáveis maternas e socioeconômicas da amostra, de acordo com a faixa etária das crianças. Santos, 2012 (n=357)

	3‐4 anos			5‐7 anos			8‐10 anos			Total	
	n	%		n	%		n	%		n	%
**Características das mães**											
*Excesso de peso pelo IMC* a											
Sim	35	41,2		69	53,5		88	61,9		192	53,9
Não	50	58,8		60	46,5		54	38,1		164	46,1
*Gordura corporal* b											
Elevada	26	30,6		34	26,9		26	18,8		86	24,6
Adequada	59	69,4		92	73,1		112	81,2		263	75,4
*Acúmulo de gordura abdominal* a											
Sim	48	56,5		79	61,2		101	71,1		228	64
Não	37	43,5		50	38,8		41	28,9		128	36

**Características de nível socioeconômico**											
*Nível socioeconômico (Abep)*											
Alto	51	60		75	58,1		86	60,1		212	59,4
Baixo	34	40		54	41,9		57	39,9		145	40,6
*Posse de carro*											
Sim	50	58,8		67	51,9		80	55,9		197	55,2
Não	35	41,2		62	48,1		63	44,1		160	44,8
*Escolaridade*											
>Ensino médio	34	40		47	36,4		35	24,5		116	32,5
≤Ensino médio	51	60		82	63,6		108	75,5		241	67,5
*Trabalha fora*											
Sim	54	63,5		76	58,9		90	62,9		220	61,6
Não	31	36,5		53	41,1		53	37,1		137	38,4

IMC, Índice de Massa Corporal; Abep, Associação Brasileira de Empresas de Pesquisa.

a n=356.

b n=349.

**Tabela 3:** Análise univariada de variáveis exploratórias e associação com acúmulo de gordura abdominal das crianças como variável dependente. Santos, 2012 (n=357)

	OR_Bruto_	IC95%	*p*-valor
**Características das crianças**			
*Sexo*			
Masculino	1,0	–	0,172
Feminino	1,4	0,8‐2,2	
*Excesso de peso pelo IMC/iade*			
Sim	62,4	30,1‐129,2	<0,001
Não	1,0	–	
*Consumo de proteína*			
Acima/igual a mediana	1,0	–	0,085
Abaixo da mediana	0,6	0,4‐1,0	

**Características das mães e nível socioeconômico**			
*Nível socioeconômico (Abep)*			
Alto	1,0	–	0,017
Baixo	0,6	0,3‐0,9	
*Posse de carro*			
Sim	1,8	1,1‐2,8	0,012
Não	1,0	–	
*Escolaridade*			
>Ensino médio	1,0	–	0,171
≤Ensino médio	0,7	0,4‐1,1	
*Trabalha fora*			
Sim	1,9	1,1‐3,0	0,010
Não	1,0	–	
*Excesso de peso pelo IMC* a			
Sim	1,7	1,0‐2,7	0,024
Não	1,0	–	
*Gordura corporal* b			
Elevada	1,9	1,0‐3,3	0,033
Adequada	1,0	–	
*Acúmulo de gordura abdominal* a			
Sim	2,2	1,3‐3,6	0,002
Não	1,0	–	

IMC, índice da massa corporal; Abep, Associação Brasileira de Empresas de Pesquisa; OR, razão de chances; IC, intervalo de confiança.

a n=356.

b n=349.

Na Tabela 4 são apresentados os resultados da análise de regressão logística e valores de *odds ratio* do modelo final, entre as variáveis estudadas. No modelo final foi considerada significativa a associação entre o excesso de gordura abdominal da criança e o excesso de peso infantil pelo IMC/idade, o sexo feminino e o acúmulo de gordura abdominal das mães. A variável de classificação socioeconômica da Abep permaneceu como variável de controle.

**Tabela 4:** Modelo de regressão logística múltipla com o acúmulo de gordura abdominal da criança como variável dependente. Santos, 2012 (n=356)

	OR_Ajustado_	IC95%	*p*-valor
*Excesso de peso pelo IMC/idade da criança*			
Sim	93,7	39,3‐223,3	<0,001
Não	1,0	–	

*Sexo da criança*			
Masculino	1,0	–	0,012
Feminino	4,1	1,8‐9,3	

*Acúmulo de gordura abdominal materno*			
Sim	2,7	1,2‐6,0	0,01
Não	1,0	–	

*Nível socioeconômico pela Abep*			
Alto	1,0	–	0,08
Baixo	0,5	0,2‐1,0	

IMC, índice de massa corporal; Abep, Associação Brasileira de Empresas de Pesquisa; OR, razão de chances; IC, intervalo de confiança.

## Discussão

No presente estudo, foram identificadas associações entre o acúmulo de gordura abdominal nas crianças e o estado nutricional da criança (excesso de peso pelo IMC/Idade), o sexo da criança (feminino) e estado nutricional da mãe (acúmulo de gordura abdominal).

Os resultados permitiram identificar uma prevalência de 30,5% de crianças com acúmulo de gordura abdominal. Estudo feito com crianças brasileiras de sete a 10 anos encontrou uma prevalência de 22% em meninas e 26,5% em meninos, valores pouco menores, mas estimados a partir de outro referencial teórico.^18^ Em outro trabalho, foi identificada uma prevalência de 33,7% de crianças com sobrepeso na cidade de Santos,^19^ valores que convergem com os dados desta investigação. Esse panorama traz que o sobrepeso e o acúmulo de gordura abdominal infantil estão presentes de forma semelhante, em relação à prevalência.

Na análise univariada, uma criança com risco de sobrepeso ou sobrepeso foi associada com apresentar acúmulo de gordura abdominal. Tal achado é consistente com o fato de que a região do tronco é uma das que apresentam maior suscetibilidade para depósitos de tecido adiposo. Apesar de essa variável ter permanecido no modelo de regressão final, é relevante considerar a superestimação do seu efeito, dada a evidente ligação do excesso de peso com o desfecho em pauta; importante também é avaliar o amplo intervalo de confiança (OR=93,7; IC95% 39,3-23,3).^12^^,^ 20 Estudo com adolescentes brasileiros produziu um resultado semelhante, ao considerar que os indivíduos com maior quantidade de gordura subcutânea teriam 133,6 mais chances de apresentar acúmulo de gordura abdominal;^21^ os autores empregaram a classificação de Taylor et al.,^12^ também adotada neste estudo.

Os resultados mostram ainda que as meninas têm 4,1 mais chances de apresentar acúmulo de gordura abdominal; espera-se encontrar maior adiposidade no sexo feminino levando em consideração o IMC;^22^no entanto, já se demonstrou associação entre adiposidade, IMC e CC em crianças e adolescentes do sexo masculino.^21^^,^ 23 Sabe-se ainda que o homem adulto apresenta maior acúmulo de gordura visceral do que a mulher;^24^ assim, vale considerar a hipótese de que a maior adiposidade abdominal encontrada nas meninas possa ser uma característica desta amostra; dito de outra forma, é possível que as condições típicas de acúmulo de gordura corporal sejam mais evidentes somente após a puberdade, ao considerar que a faixa etária da amostra pode ser muito jovem.

As atividades consideradas como práticas sedentárias não mostraram associação com o acúmulo de gordura abdominal, o que contraria achados pregressos sobre o efeito do tempo de assistir a TV e do transporte ativo até a escola.^10^^,^ 22 ^,^ 23 Um estudo com adolescentes brasileiros também encontrou resultado contrário: um nível insuficiente de prática de atividade física foi associado com uma CC elevada.^25^ É possível que as variáveis em estudo não tenham sido bons marcadores de comportamentos sedentários, que podem trazer riscos a saúde nessa população. No entanto, deve-se considerar que o tempo gasto assistindo a TV e usando computador era relatado pela mãe da criança e pode ter sido subestimado.

O consumo de proteína elevado mostrou associação com acúmulo de gordura abdominal, na análise univariada, e perdeu posteriormente significância no modelo de regressão. O acréscimo de proteína na dieta apresentou relação contrária: por meio de mudanças na dieta, mais proteína gerou uma redução de 2,7 cm da CC em crianças e adolescentes, em relação a grupo com pouca proteína, em estudo longitudinal.^26^

O estado nutricional materno apresentou associação significativa com a gordura abdominal na criança em todos os parâmetros avaliados e, principalmente, com a medida de CC na análise multivariada. Em nossa amostra, uma mãe com acúmulo de gordura abdominal aumenta em 2,7 vezes a chance da criança também apresentar esse quadro. Pesquisadores no México relatam que filhos de pais com obesidade abdominal têm 2,85 mais chances de apresentar o mesmo quadro,^9^ valor próximo ao encontrado neste estudo. Outras investigações também encontraram associação entre sobrepeso da criança e obesidade materna.^22^^,^ 23 Isso indica que tanto o estado nutricional da mãe quanto o do pai da criança podem ter relação com esse desfecho. Deve-se considerar que tal influência possa estar associada tanto a fatores genéticos como socioculturais de hábitos familiares. O cuidado com a atenção nutricional à saúde materno-infantil, portanto, deve se iniciar no período pré-natal e considerar toda a estrutura familiar. Já se demonstrou que a participação dos pais em intervenções de educação nutricional e promoção de atividade física para crianças auxilia beneficamente na redução do IMC e outros parâmetros do estado nutricional.^27^Deve-se considerar previamente que a percepção dos pais em relação ao estado nutricional de seu filho pode ser uma barreira, tendo em vista a dificuldade na identificação do excesso de peso e, portanto, no reconhecimento da importância de incluir seu filho em tais atividades.^28^

Embora não tenha apresentado significância no modelo final, a análise univariada mostrou associação significativa entre o acúmulo de gordura abdominal das crianças e o nível socioeconômico pela estratificação social, a posse de carro e o fato de a mãe trabalhar fora de casa. Nível socioeconômico elevado, representado pelo tipo de escola e cidade, também já foi associado com acúmulo de gordura abdominal, em crianças indianas.^8^ Contudo, outro estudo com crianças brasileiras menores de cinco anos não observou associação entre o nível socioeconômico, também representado pela Abep, e excesso de peso;^29^ pode-se sugerir que há outros fatores com maior influência sobre o estado nutricional do que o nível socioeconômico.

A posse de carro poderia também estar associada com a atividade física. As crianças cujos pais apresentam esse bem material o usariam para as atividades diárias, o que evita momentos de caminhada ou uso de bicicleta, por conveniência ou violência urbana.^10^ Quanto às crianças cujas mães trabalham fora, tais mães, pela impossibilidade de estar presente durante o dia para preparar uma refeição balanceada ao filho, acabam por optar por opções convenientes e rápidas para garantir alguma alimentação aos filhos, hábitos que podem ser incorporados pelas crianças posteriormente.

O estudo da ingestão alimentar apresenta limitações como número reduzido de recordatórios de 24 horas e a obtenção do dado por meio de entrevista com a mãe, o que pode determinar subnotificação. Além disso, tratando-se de um estudo que aplicou um questionário de investigação extenso, algumas perguntas e medidas não puderam ser aplicadas ou aferidas pelos entrevistadores por alegado desconforto ou recusa do entrevistado e, assim, o *n* de algumas variáveis é menor. A taxa de resposta de 70% também pode ter interferido nos resultados de algumas associações. Ademais, como se trata de estudo transversal, não é possível relatar relação de causalidade entre os fatores analisados. Apesar dessas limitações, como contribuições do presente estudo, constatou-se que o estado nutricional materno, a ocorrência de excesso de peso e o sexo feminino em crianças estão associados com o acúmulo de gordura abdominal em crianças, independentemente de seu nível socioeconômico.

A síndrome metabólica em crianças ainda é um tema emergente, para o qual diferentes aspectos clínicos são considerados no diagnóstico, a depender do seu referencial teórico.^30^ A CC elevada constitui um desses aspectos e, ao comparar com outros, como níveis séricos de glicose e lipídios, apresenta vantagens por ser um procedimento não invasivo e de fácil mensuração. Assim, sua inclusão na rotina da atenção básica à saúde auxiliaria no delineamento de intervenções e atividades interdisciplinares ligadas ao combate de doenças crônicas na infância.

As relações que envolvem a CC da criança ainda são incertas. Mais estudos nessa linha de pesquisa são necessários para comprovação de algumas das hipóteses aqui apresentadas. Espera-se que este estudo contribua para o desenvolvimento de políticas públicas de prevenção de doenças crônicas, ao considerar a importância de ações de educação nutricional concebidas para o âmbito familiar, de forma a resultar em maior chance de impacto sobre o estado nutricional das crianças.
